# Medico-Chirurgical Society of Montreal

**Published:** 1880-07

**Authors:** 


### MEDICO-CHIRURGICAL SOCIETY OF MONTREAL.

The regular meeting was held April 16th, 1880, the President in the chair.

Dr. James Cameron read a paper on the communicability of typhoid fever by milk, in Montreal, with special reference to one dairy on the Lachine Road. This dairy supplies some thirty families in the city, besides some large institutions, including the St. Lawrence Hall. It was ascertained by examination that out of the thirty families supplied by this milkman, thirteen of them had had typhoid fever since October, 1879. One notable feature connected with these cases was, that a number proved fatal, while those that finally recovered had repeated relapses. A case of typhoid fever had occurred at this milkman's house, prior to this outbreak. The well out of which water was taken for the dairy use, was situated in a manner adjacent to the water-closet, so that the excreta placed there could easily be conveyed from one to the other. The quantity of milk supplied to the St. Lawrence Hall, was about twenty-five gallons a day.

Dr. F. W. Campbell said that he had had one case of typhoid fever at the St. Lawrence Hall, in October, a severe case, which had proved fatal. This source of typhoid was not a new one, yet newer than many of us think. It is a remarkable thing that not a single one of the latest and best text books (excepting Roberts') mentions impure milk, or milk contaminated with typhoid germs, as a cause of the disease. Dr. Campbell mentioned that remarkable outbreak that occurred in a certain district in London, in 1874, where the infected family of the late Dr. Murchison were and where that medical man, after careful and persistent efforts, discovered the source of the trouble.

Dr. Reddy said six out of the cases mentioned by Dr. Cameron were under his care. Very severe relapses were noticeable in these cases. He had now four fresh cases in families supplied by the same milkman.

Dr. Girdwood said that he had water from this well in process of examination, but could not at present make a report on it. The position of the well from which the cattle were supplied, was such as to be easily contaminated from the drainage of the yard.

Dr. Osler said there are two points worthy of attention. There would appear here to be a continuous source of infection, extending over six months, in that it differs from recorded cases where the outbreak has been simultaneous and within a limited area. The second point was, whereas thirteen out of thirty families were affected, yet only one case is recorded in the St. Lawrence Hall.

Dr. Ross suggested the advisability of a more carefully prepared statistical report, in order to arrive at the true state of the case.

Dr. Hy. Howard said much of the city milk was brought from Longue Pointe. The cattle were watered from holes in the ice, and water was taken from this same source for household and dairy purposes. The water of the river in that vicinity must be much contaminated by the refuse and drainage of the city, and doubtless is also a cause of the propagation of this disease.

Dr. R. P. Howard expressed the indebtedness of the Society to Dr. Cameron for the evidence supplied on this subject, but there are so many points that arise, that Dr. Cameron would do well to further investigate these cases, especially as to the St. Lawrence Hall. This discussion would, doubtless, cause more attention to be given to the subject.

Mr. McEachran introduced for discussion "The Transmissibility of Tuberculosis from Animals to Man," particularly by milk, with the object of impressing the importance of regular inspection of the cattle in the dairies supplying the city with milk, as well as of the dairies themselves.

The experiments of continental investigators have so clearly established the close relationship between tuberculosis in man and in animals, and the possibility of the disease being in many ways transmitted from one to the other, that the guardians of the public health would be perfectly justified in using every precaution against milk of tuberculous cows being sold for consumption.

He pointed out the necessity, also, of providing some means whereby proper scientific experiments could be conducted to test the statements of these foreign scientists, for at present we have but little else to guide us.

He described the experiments and different views of the French and German pathologists Villemin, Chauveau, St. Cry, Viseur, Klebs, Orth, Collinger, and others, which adduced sufficient evidence of the transmissibility of this disease, by ingestion, inoculation, and inhalation of the virus, and particularly the danger from the use of milk from phthisical cows, to warrrnt the Society in passing resolutions recommending precautionary measures on the part of the Board of Health.

Dr. R. P. Howard, in opening the discussion, remarked that although the members of the Society were aware that the transmission of tuberculosis from animal to animal by milk, and possibly to the human subject, had, within late years, engaged the attention of scientific men, we were pleased that Mr. McEachran had brought the matter formally before us, no doubt with the view of learning the opinions of the members upon it. As Mr. McEachran's paper consists mainly of extracts from Dr. Vallin's recent article entitled, "Can the milk of phthisical cows produce tuberculosis," and advances no opinion of his own, one may venture to call in question very frankly the hasty conclusions which have been drawn by a few experimentalists in France and Germany, not agreed amongst themselves, upon so difficult and important a topic.

Before stating his objections to the inferences that have been drawn respecting the communication of tuberculosis from experiments upon the lower animals, he would remind the Society that these experiments claim to have proved that the inhalation of sputum from phthisical cavities, of simple bronchitic sputum, of particles of cheese, of pulverized brain, or even of cinnabar by dogs, rabbits and other animals, produces tuberculosis of the lungs, and that the *ingestion* of phthisical sputa, of catarrhal sputa, of common pus, and of the milk of phthisical cows likewise produces tuberculosis in the lower animals.

Bearing these alleged results of experiment in mind, one may fairly object to the doctrine that the milk of phthisical cows transmits tuberculosis—

1st. That the resulting lesions cannot be attributed to any *specific* tuberculous nature of such milk, inasmuch as such unlike substances as phthisical sputum, catarrhal expectorations, ordinary pus, cheese and cinnabar produce the same lesions in these animals.

In one of Kleb's cases even *boiled* milk produed the same consequences as the same milk not boiled, and Zurn found that boiling tuberculous matter did not render it innocuous to the animal that ingested it. A boiling temperature, it is generally believed, will destroy the germs of the specific morbid poisons.

It is too wide and difficult a subject, even if it were necessary to my argument, to attempt an explanation of these results of experiments upon animals; and my

2nd. Point is, that even were it established that the milk of phthisical cows, when ingested, produces tuberculosis in calves and other animals, that would not warrant the statement that such milk would have a like effect if ingested by the human subject.

3rd. Every animal has an organization proper to itself, and its own aptitudes and endowments as regards diseased actions. It must be shown—proved, not guessed—that the milk of tuberculous cows, drank by human beings, will impart tuberculosis to them, before it can be accepted as a fact.

4th. The irritation caused by a simple wound, by a seton of silver wire, or cotton, or paper, by the introduction of pus, cinnabar, etc., under the integuments, produces the so-called artificial tuberculosis in guinea-pigs, rabbits, and other animals; but what pathologist now maintains that such irritation produces tuberculosis in man?

5th. It has not yet been proven that tubercle itself is directly innoculable in the healthy human subject, although it may be admitted that when tubercle has been developed in the human body it may enter the lymphatics or blood-vessels, and be thus propagated in near or in remote parts.

6th. It is not yet agreed amongst pathologists that in the human subject phthisis is contagious or communicable from person to person, although much may be said on the affirmative side of of the question, and Weber's cases are very important. But even if phthisis be communicable through the semen, as syphilis is thought by some to be, fecundation and gestation are so essentially peculiar processes, that no analogical argument from them can, in the present state of knowledge, be safely applied to the explanation of diseases which are contagious or communicable in the ordinary modes.

7th. No reliable instance in which the milk of a phtisical cow has induced tuberculosis in the human subject is recorded. Even Bollinger, in his latest paper on this subject, claims to know of but one case, and he is frank enough to admit that it is only probable that the child had been fed upon the milk of a tuberculous cow.

8th. The circumstance that infants and children are not specially obnoxious to tuberculous disease, although their food is chiefly, and for the first year almost solely, milk, is strongly opposed to the theory in question.

When it has been proved that the milk of phthisical cows does induce tuberculosis in man, several questions will require solution,—such as, (1) Under what circumstances does such milk produce the disease? (2) Can it do so on healthy persons free from tuberculous or scrofulous predisposition ? (3) If it can, is it from any quality, and if so, what quality, peculiar to such milk? (4) May other milk than that from tuberculous cows induce tuberculosis?

Such are some of the objections that, in my opinion, have to be satisfactorily met before medical men can properly affirm that the milk of phthisical cows will produce tuberculosis in human beings. But the subject is one of such great importance, and the interests at stake so serious, that until it can be shown that the theory in question is quite erroneous, we shall feel it our duty, as a Society, to urge upon the authorities the wisdom of establishing an efficient system of inspecting not only the milk supplied to the citizens, but the sanitary state of the cows supplying the milk, and, indeed, the condition of the daries also.

Dr. Osler said the views expressed by the President were substantially the views of pathologists of the present day. Few animals have been experimented on. The subject is surrounded by many difficulties. It is notorious how many of these thorough-bred cattle have inflammation of the lungs, which ends with caseation, and this is reckoned by all veterinary surgeons as phthisis. The chief end of this discussion should result in a proper milk inspection, not only of the dairies, but of the milk brought into the city. In Berlin, a double set of officers are appointed to all the roads leading into that city, whose duty is to examine the milk, and good has resulted both there and in Paris.

The President nominated Drs. Osler, Ross and Larocque a committee to draw up a motion embodying the views of the Society on the subject of a pure milk supply, to be presented to the City Council.

---

The regular meeting was held April 30th, the President in the chair.

On the part of the committee appointed at the last meeting, Dr. Ross moved, seconded by Dr. Osler, the following resolution:

"Whereas, it is universally admitted that a supply of pure milk is of paramount importance to the well-being of the community, particularly the infantile population; and whereas, it has been amply proven that various diseases may be caused by, or communicated through, unwholesome or impure milk,—*Resolved*, That the Secretary be instructed, on the part of this Society, to urge upon the Board of Health the necessity of enforcing the existing By-Laws concerning the inspection of dairies and the examination of milk-giving cattle and their surroundings by a competent Veterinary Surgeon, who should report at regular intervals to the health officer of the city."

Dr. Ross read the by-laws, and said that in considering the matter the committee had concluded that, in almost all essential matters, the by-laws of the city already made ample provision for the enforcement of milk inspection, but that they had never been acted upon, and had, in fact, been allowed to become a complete dead letter. The latter part of the resolution also alluded to another very important matter for which no arrangements were made by the existing regulations. The President thought that greater stress should be laid upon non-enforcement of by-laws and an absence of proper milk inspection for the city. One officer should do the work. A Veterinary Surgeon should inspect the milk as well as the surroundings of the cattle and also the relations of privies to wells, and such like sanitary matters. The health officers would no doubt gladly delegate this to another officer. After remarks by several other members, the resolution was put and unanimously carried.

Dr. Osler presented to the Society a case of peculiar heart-murmur. The special feature here presented was that, in the ordinary position, sitting or standing, no murmur was audible, but on bending slightly forward a loud musical murmur was at once developed. This varied in intensity at different times, but sometimes was loud enough to be heard several feet away from the patient. These facts were verified by all the members present. There was no hypertrophy and no other evidence of cardiac disease. The sound and the position of the girl's body in which it was loudest, had been noticed by herself and her friends. Ebstein mentions several cases of very loud murmurs heard at a distance, most of them connected with mitral stenosis; only three in which there was no disease. Dr. Osler cannot account for the murmur, but thinks, perhaps, it arises from an unusually flexible chest and pressure thus exerted on the pulmonary artery and aorta. Dr. Ross suggested that it might possibly be explained by the existence of some loosely pedunculated body hanging inside the heart in such a way that, only in certain positions it would fall into the blood-current and develop a murmur. At all other times, of course, there was none. He had had a patient last year in the general hospital in whom there was a loud apex murmur when sitting up, none at all lying down. The President rather favored the idea that it was most likely due to bending of the costal cartilages and pressure on the pulmonary artery.

Dr. R. P. Howard read a paper upon a case of fibroid degeneration of the heart.

Dr. Osler said that he had only met with one other case, although the apex of the papillary muscles are often affected with fibroid degeneration. A very common condition is the development of milky patches in the pericardium, but here the fibroid change does not extend into the subjacent muscle.

The meeting then adjourned.—*Canada Medical Journal.*

